# Circulating fibroblast growth factor 21 levels in gestational diabetes mellitus and preeclampsia: a systematic review and meta-analysis

**DOI:** 10.1186/s12884-025-07157-3

**Published:** 2025-01-16

**Authors:** Zhen Cao, Zhiming Deng, Jieyi Lu, Ying Yuan

**Affiliations:** 1 Department of Laboratory Medicine, Zhujiang Hospital, Southern Medical University, Guangzhou, Guangdong 510260 China; 2 Department of Clinical Laboratory, Guangdong Provincial Key Laboratory of Major Obstetric Diseases, Guangdong Provincial Clinical Research Center for Obstetrics and Gynecology, The Third Affiliated Hospital of Guangzhou Medical University, Guangzhou, 510150 China; 3 Department of Clinical Laboratory, The Third Affiliated Hospital of Guangzhou Medical University, 63 Duobao Road, Liwan District, Guangzhou, Guangdong China

**Keywords:** Gestational diabetes mellitus, Preeclampsia, Fibroblast growth factor 21, Meta-analysis, Diagnosis

## Abstract

**Background:**

The connection between fibroblast growth factor 21 (FGF21) and the likelihood of gestational diabetes mellitus (GDM) or preeclampsia (PE) has received more attention recently. Based on published articles, meta-analysis were conducted to explore the differences in FGF21 levels in GDM or PE compared to control groups.

**Methods:**

Articles published before April 5, 2024 were searched across four databases: PubMed, Web of Science, Embase, and Cochrane Library, and studies exploring the association of FGF21 levels and GDM or PE were collected. Additionally, ClinicalTrials.gov was also searched for completed and ongoing trials. (Prospero Registration CRD42024504738). The standardized mean differences (SMDs) and 95% confidence intervals (CIs) were utilized to determine FGF21 levels among different groups.

**Results:**

This analysis incorporated a total of 16 articles, with 714 GDM and 701 non-GDM in the control group. The GDM-affected pregnant women had greater levels of circulating FGF21 than the control group (SMD = 0.529, 95% CI: 0.168 ~ 0.890, *p* = 0.004). Moreover, the PE case group covered 120 while the control group contained 134. The findings indicated that pregnant women with PE had significantly greater levels of circulating FGF21 than healthy expectant mothers (SMD = 0.743, 95% CI: 0.527 ~ 0.958, *p* = 0.000).

**Conclusions:**

Our study found that FGF21 has the potential to serve as a diagnostic marker for GDM or PE. However, due to the limited number of studies and the fact that most data were from the second and third trimesters of pregnancy, more large-scale prospective studies are needed to validate these conclusions, investigate the potential of FGF21 in enabling early diagnosis, and further examine the role of FGF21 in the development and progression of GDM/PE.

**Trial registration:**

Not applicable.

**Supplementary Information:**

The online version contains supplementary material available at 10.1186/s12884-025-07157-3.

## Background

As a prevalent pregnancy complication, gestational diabetes mellitus (GDM) is becoming a significant public health concern as the incidence of obesity throughout the world grows, endangering the health of millions of women and their offspring [1]. Globally, GDM affects 2% ~ 38% of pregnant women. However, global prevalence differs according to the sample population and diagnostic criteria, with the average prevalence of about 14.8% in China [2–4]. Adverse pregnancy outcomes, especially, premature labor, macrosomia, hypoglycemia in neonates, infant jaundice and neonatal respiratory distress syndrome, have been linked to GDM. Over time, it also elevates the risks of type 2 diabetes mellitus, obesity and cardiovascular diseases in women and children [3, 5–7]. Currently, an oral glucose tolerance test (OGTT) is generally done between weeks 24 and 28 of gestation to diagnose GDM, which is not beneficial to early intervention in high-risk pregnant women. As the incidence of GDM increases, exploring novel diagnostic biomarkers for GDM is still imperative to promote diagnosis, especially early diagnosis.

Preeclampsia (PE) affects about 3% ~ 5% of pregnant women, which can result in serious damage to their liver, brain, kidneys and other organs. As one of the primary causes of maternal mortality, it is often associated with fetal growth restriction, premature delivery and fetal distress [8, 9]. The diagnosis of PE is confirmed by signs of hypertension and proteinuria that appear after 20 weeks of pregnancy, while its sensitivity and specificity are far from satisfactory. Consequently, new diagnostic biomarkers for PE have become a research focus in recent years, intending to find a biomarker that could improve outcomes for mothers and newborns by providing a definitive diagnosis and monitoring the disease progression.

Expressed in the adipose tissue, liver, placenta and pancreas, fibroblast growth factor 21 (FGF21), a key modulator of energy metabolism, is involved in glycolipid metabolism and other metabolic processes [10, 11]. At present, a few studies have sought to investigate the differences in circulating FGF21 levels between pregnant women with GDM/PE and healthy pregnant women. However, considering the limited sample size of a single study involved and the different results yielded, we performed a meta-analysis to gain a deeper knowledge of the relationship between circulating FGF21 levels and the likelihood of GDM and PE, offering more compelling evidence for the diagnostic value of FGF21 in GDM and PE.

## Methods

### Search strategies and selection criteria

The current study was registered on the International Prospective Register of Systematic Reviews, with registration number CRD42024504738. The Preferred Reporting Items for Systematic Reviews and Meta-Analyses (PRISMA) Statement was followed in the reporting of the systematic review and meta-analysis.

For collecting relevant English articles published before April 5, 2024, four databases were searched: PubMed, Web of Science, Embase and Cochrane Library. The following combined text and MeSH terms were employed: “FGF21” and (“Diabetes, Gestational” or “Pre-Eclampsia”). The comprehensive list of PubMed’s retrieval formulas is provided in Supplementary material 1. Articles were screened by two authors independently (ZC, ZD), and any differences were settled with the help of the third author (YY). To broaden the scope of retrieval, ClinicalTrials.gov was also searched for finished and current trials, and the references of published studies were screened.

### Study selection and data extraction

Inclusion criteria were as follows: (1) The study was composed of two groups, the control group included healthy expectant mothers with no pregnancy complications, and the case group included GDM-affected pregnant women or PE; (2) The study population was pregnant women without pre-existing diabetes before pregnancy; (3) The study evaluated the differences in circulating FGF21 levels between the case and control groups; (4) The circulating FGF21 level was used as an outcome measure in data analysis; (5) No restriction on types of articles (including cohort studies, case-control studies, RCTs); (6) Articles in the English language.

Exclusion criteria were as follows: (1) Studies included patients with prior diabetes; (2) The diagnosis of the case group in the study was not clear enough, for example, patients with gestational hypertension instead of PE were included; (3) The studies did not focus on GDM or PE to evaluate its differences with the control group in FGF21 levels; (4) Studies combining FGF21 with other biomarkers; (5) Studies with incomplete content, missing data or unextractable data; (6) Studies on non-human subjects: genes, animals, or cells; (7) Uncontrolled studies; (8) The levels of FGF21 measured were not those of circulating FGF21, such as those found in amniotic fluid or placental tissue; (9) Case reports, expert opinions, reviews, conference abstracts, patents and letters to the editor; (10) Duplicates or articles published in languages other than English.

Two researchers (ZC, ZD) independently retrieved and screened articles according to predefined inclusion criteria, and then extracted data after detailed analysis. Any differences were settled with the help of the third researcher (YY). Name of the first author, year of publication, nation where the study was performed, diagnostic criteria for GDM/PE, sample size of the case group/control group, age, the time of sample collection, testing methods for FGF21, the mean and standard deviation (SD) of FGF21 and article quality rating according to the Newcastle-Ottawa scale (NOS) were among the primary data extracted. NOS rating scale was provided in Supplementary material 2. Studies scoring 0 to 3 were considered poor quality, those scoring 4 to 6 were considered fair quality, and those scoring 7 to 9 were considered good quality [12].

### Statistical analysis

The FGF21 level was analyzed as a continuous variable in statistical analyses, which was reported as mean and SD. Data expressed as median and interquartile range were converted to mean and SD [13, 14]. As the units of effect size varied in included studies, the standardized mean differences (SMDs) and 95% confidence interval (CI) were utilized as the pooled effect size to compare FGF21 levels in pregnant women in good health to those who have PE or GDM. Mata-analyses were conducted using Stata 15.0, and the heterogeneity among the incorporated studies was evaluated with Cochran’s Q test and *I*^*2*^ index. Specifically, if the results were heterogeneous, the effect size was combined using a random-effects model (*p* < 0.05, *I*^*2*^ > 50%), but if the homogeneity was high, a fixed-effects model was employed to combine the effect size (*p* ≥ 0.05, *I*^*2*^ ≤ 50%). To further investigate the connection between FGF21 levels and the likelihood of GDM/PE, subgroup analyses were conducted according to the time of sample collection, region, sample size, diagnostic criteria and quality of included studies. The sources of heterogeneity were investigated through sensitivity analyses. Sensitivity analyses were conducted by excluding each study separately, and the effect sizes of the remaining studies added together were computed. The total effect size and publication bias were evaluated using forest plots and funnel plots. Moreover, Begg’s and Egger’s tests and funnel plots were employed to examine the presence of publication bias, with significant publication bias defined as *p* < 0.05.

## Results

**Fig. 1:**
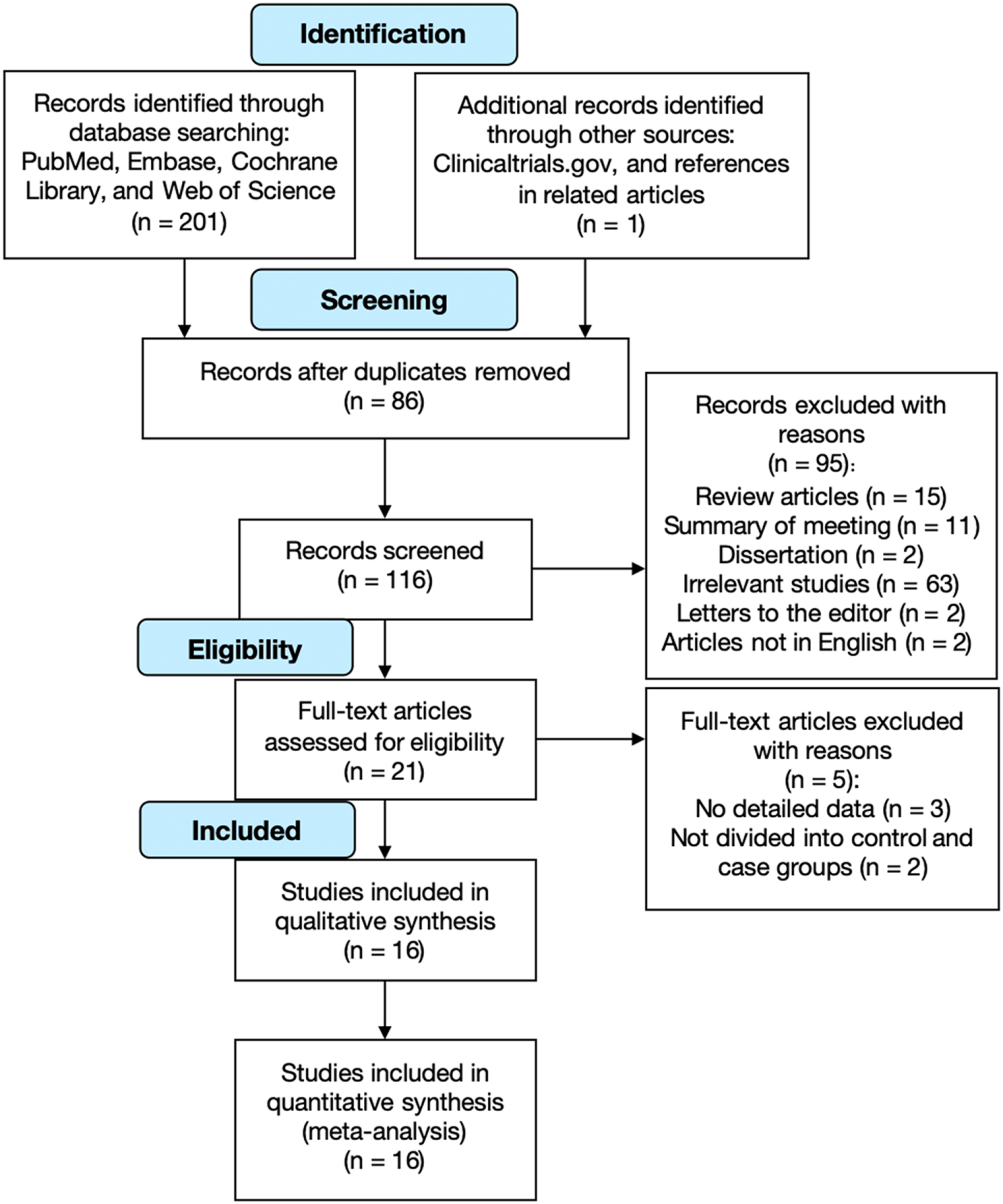
Flow chart of literature selection

A total of 202 articles were retrieved from databases, and the complete retrieval formulas of PubMed are detailed in Supplementary material 1. Among them, 116 studies were left after duplicates were removed. Following the reviewing of titles as well as abstracts, 95 articles were chosen for full-text evaluation. Ultimately, this study included 16 articles. The literature screening process is depicted in Fig. 1.

Table 1 summarizes the fundamental features of these 16 studies. The selected studies were case-control studies and cohort studies based on hospital populations, and were published between 2010 and 2023. Among them, there were 10 articles with a NOS score of 8, 4 articles with a score of 7, and 2 articles with a score of 6. Of the 16 studies, 13 were related to GDM and 3 were related to PE. They covered data from eight countries: 2 from Germany, 2 from Spain, 7 from China, 1 from Australia, 1 from Iran, 1 from Malaysia, 1 from Colombia and 1 from Poland. Furthermore, 1,669 pregnant women were included in all, consisting of 714 patients with GDM, 120 patients with PE and 835 healthy expectant mothers.

**Table 1:** Characteristics of available studies relating FGF levels to GDM or PE risk

Number	Author	Year	Country	Participants (cases/controls)	Age(cases/ controls)	Disease	Diagnostic criteria	Time of FGF21 measurement	Circulating levels of FGF 21 (pg/mL)	Assay method	Study quality (NOS)
1	Sebastian Stein	2010	Germany	40/80	33 ± 10/28 ± 5	GDM	the Austrian Diabetes Association	205 ± 30/ 198 ± 39 days of gestation	97.5 ± 123.04/102.9 ± 107.33	ELISA	fair
2	Holger Stepan	2013	Germany	51/51	30 ± 11/30 ± 7	PE	ISSHP	208 ± 37/197 ± 26 days of gestation	309.6 ± 361.19/105.2 ± 148.07	ELISA	good
3	Bee K. Tan	2013	Spain	12/12	34.42 ± 7.13/ 32.17 ± 5.03	GDM	WHO	39–40 weeks of gestation	246.86 ± 169.82/122.2 ± 107.42	ELISA	good
4	Dongyu Wang	2013	China	30/60	29.33 ± 3.29/ 28.95 ± 3.14	GDM	IADPSG	25.37 ± 1.25/25.23 ± 1.05 weeks of gestation	132.77 ± 83.12/71.82 ± 36.67	ELISA	good
5	Lu Xu	2013	China	120/60	31.65 ± 3.75/ 31.21 ± 5.32	GDM	ACOG	24–28 weeks of gestation	375.81 ± 525.26/441.3 ± 607.67	ELISA	good
6	Marloes Dekker Nitert	2014	Australia	10/10	NA	GDM	Australasian Diabetes In Pregnancy guidelines	38–39 weeks of gestation	452.33 ± 727.56/358.42 ± 586.52	ELISA	fair
7	Si-ming Li	2015	China	51/50	30.53 ± 20.78/29.42 ± 18.39	GDM	IADPSG	28th week of pregnancy	400.04 ± 914.46/247.62 ± 757.31	ELISA	good
8	Ana Megia	2015	Spain	79/78	32.15 ± 5.09/30.82 ± 4.78	GDM	Spanish diabetes in pregnancy guidelines	26–29 weeks of gestation	107.89 ± 77.15/85.62 ± 80.15	ELISA	good
9	Shokoufeh Bonakdaran	2017	Iran	30/60	26.8 ± 6.3/24.8 ± 4.3	GDM	IADPSG	24–28 weeks of gestation	264.5 ± 196.2/59.1 ± 36.5	ELISA	good
10	Dongyu Wang	2019	China	20/25	29.16 ± 3.43/28.72 ± 3.11	GDM	the Ministry of Health China in 2014	38.91 ± 1.03/39.12 ± 1.01 weeks of gestation	199.31 ± 225.84/156.81 ± 187.27	ELISA	good
11	Maryam Mosavat	2020	Malaysia	53/43	33.2 ± 0.6/32.1 ± 0.8	GDM	ADA	24–28 weeks of pregnancy	200.2 ± 247.52/255.2 ± 217.71	magnetic bead-based mul-tiplex immunoassay	good
12	Lin Jiang	2020	China	49/31	29.02 ± 4.28/ 29.94 ± 4.19	PE	ISSHP	within 2 days before delivery as close as possible	462.53 ± 188/366.85 ± 191.49	ELISA	good
13	Zhiheng Wang	2021	China	133/133	29.53 ± 2.27/ 29.54 ± 2.62	GDM	IADPSG	14–21 weeks of pregnancy	66.04 ± 53.73/39.45 ± 32.96	ELISA	good
14	Julieth Daniela Buell-Acosta	2022	Colombia	20/52	23.6 ± 5.3/25.6 ± 6.3	PE	ACOG	First-trimester	105.84 ± 66.32/67.86 ± 31.76	ELISA	good
								Second-trimester	119.65 ± 91.96/78.37 ± 46.67		
								Third-trimester	303.24 ± 279.325/133.97 ± 84.56		
15	Xiaojiao Jia	2022	China	62/58	29.38 ± 4.65/28.93 ± 3.31	GDM	IADPSG	24–28 weeks of gestation	192.07 ± 40.26/117.33 ± 33.8	ELISA	good
16	Katarzyna Gawlik	2023	Poland	74/32	29.7 ± 4.0/ 28.5 ± 2.5	GDM	WHO	second trimester	92.6 ± 87.03/70.91 ± 88.62	ELISA	good

ISSHP, International Society for the Study of Hypertension in Pregnancy; WHO, World Health Organization; IADPSG, International Association of Diabetes and Pregnancy Study Group; ACOG, American College of Obstetriciansand Gynecologists; ADA, Americn Diabetos Association; ELISA, enzyme- linked immunosorbent assay; NOS, Newcastle Ottawa Scale

**Fig. 2:**
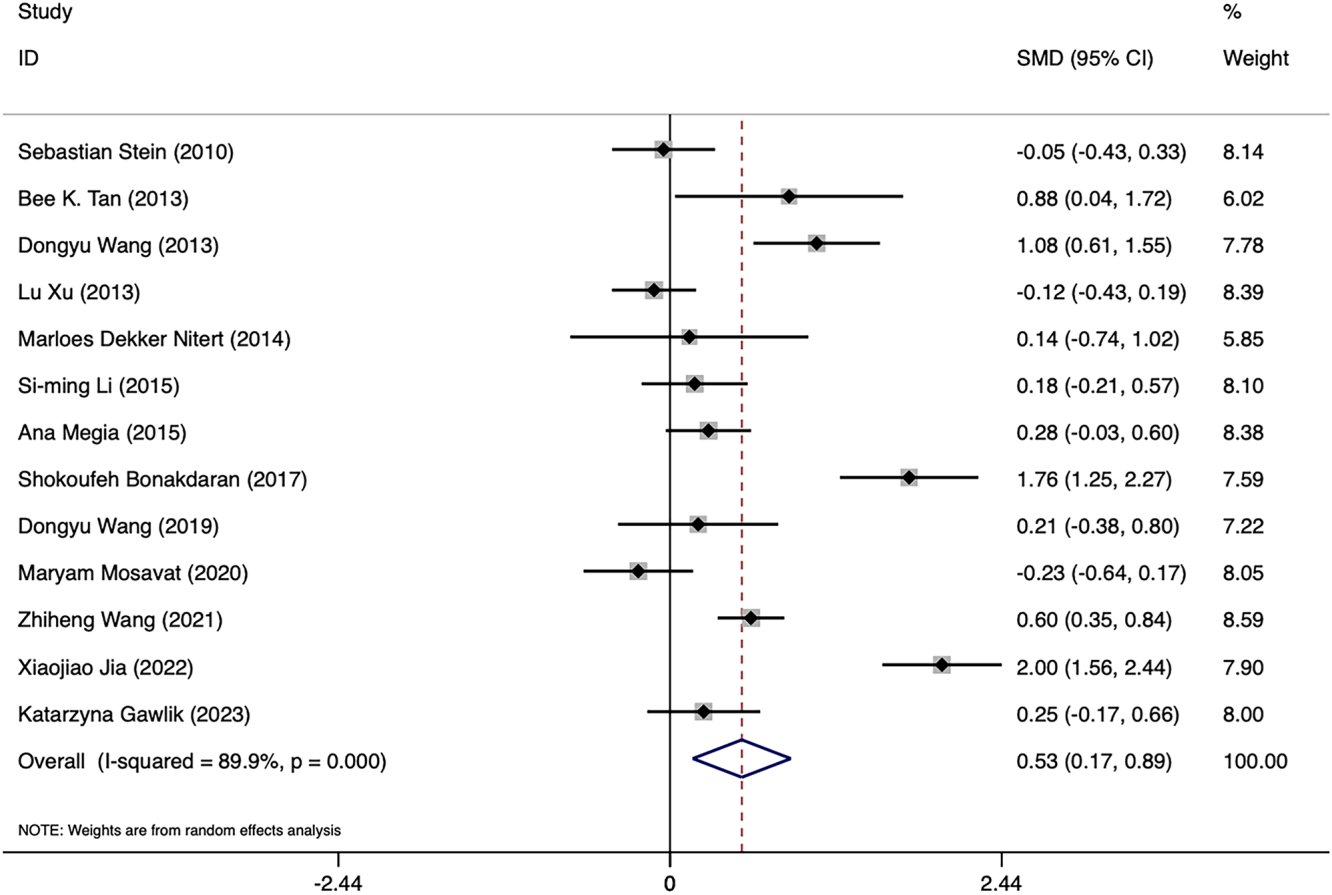
Forest plot of meta-analysis of the relationship between FGF21 levels and GDM. SMD: Standard Mean Difference; CIs: Confidence Intervals

**Fig. 3:**
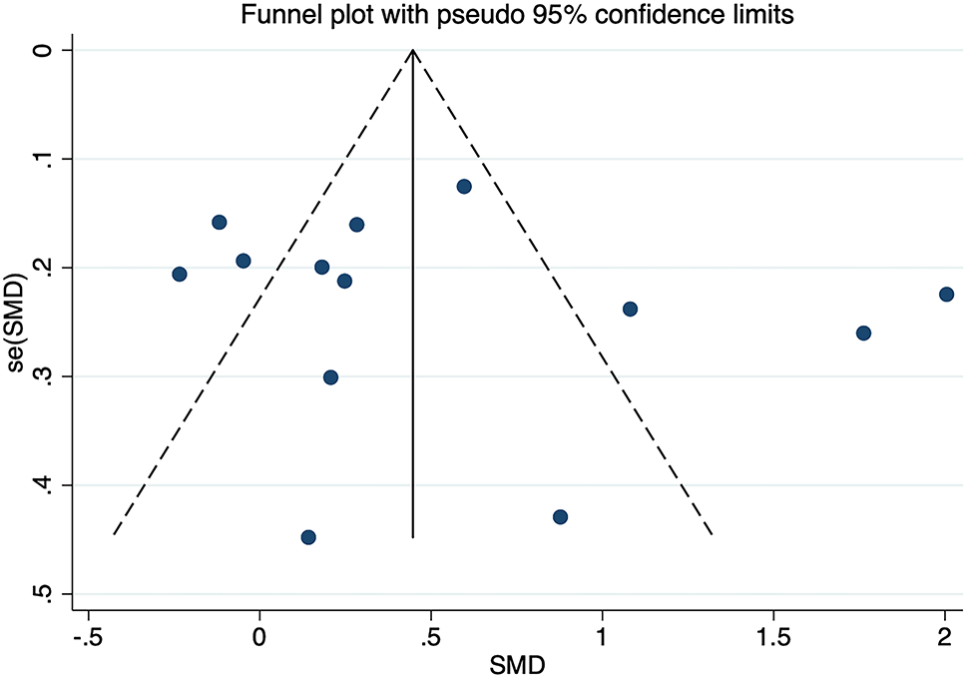
Funnel plot for publication bias of FGF21 levels and GDM. SMD: Standard Mean Difference; se(SMD): Standard Error of Standard Mean Difference

Substantial heterogeneity was found among the 13 studies pertaining to GDM, which comprised 714 GDM-affected pregnant women and 701 healthy expectant mothers (*I*^2^ = 89.9%, *p* = 0.000) (Fig. 2). Data were analyzed using a random-effects model, and results displayed that a significant increase in circulating FGF21 levels was observed in GDM-affected pregnant women in comparison to healthy expectant mothers (SMD = 0.529, 95% CI: 0.168 ~ 0.890). The differences were statistically significant (*p* = 0.004). Sensitivity analyses were carried out by excluding one study at a time, and the results demonstrated that none of the individual studies significantly changed the effect size. Begg’s and Egger’s tests were adopted to determine whether publication bias existed, and results indicated that the funnel plot was approximately symmetrical, suggesting that studies included in the meta-analyses did not exhibit any notable publication bias (*t* = 0.74, *p* = 0.476), as shown in Fig. 3.

**Fig. 4:**
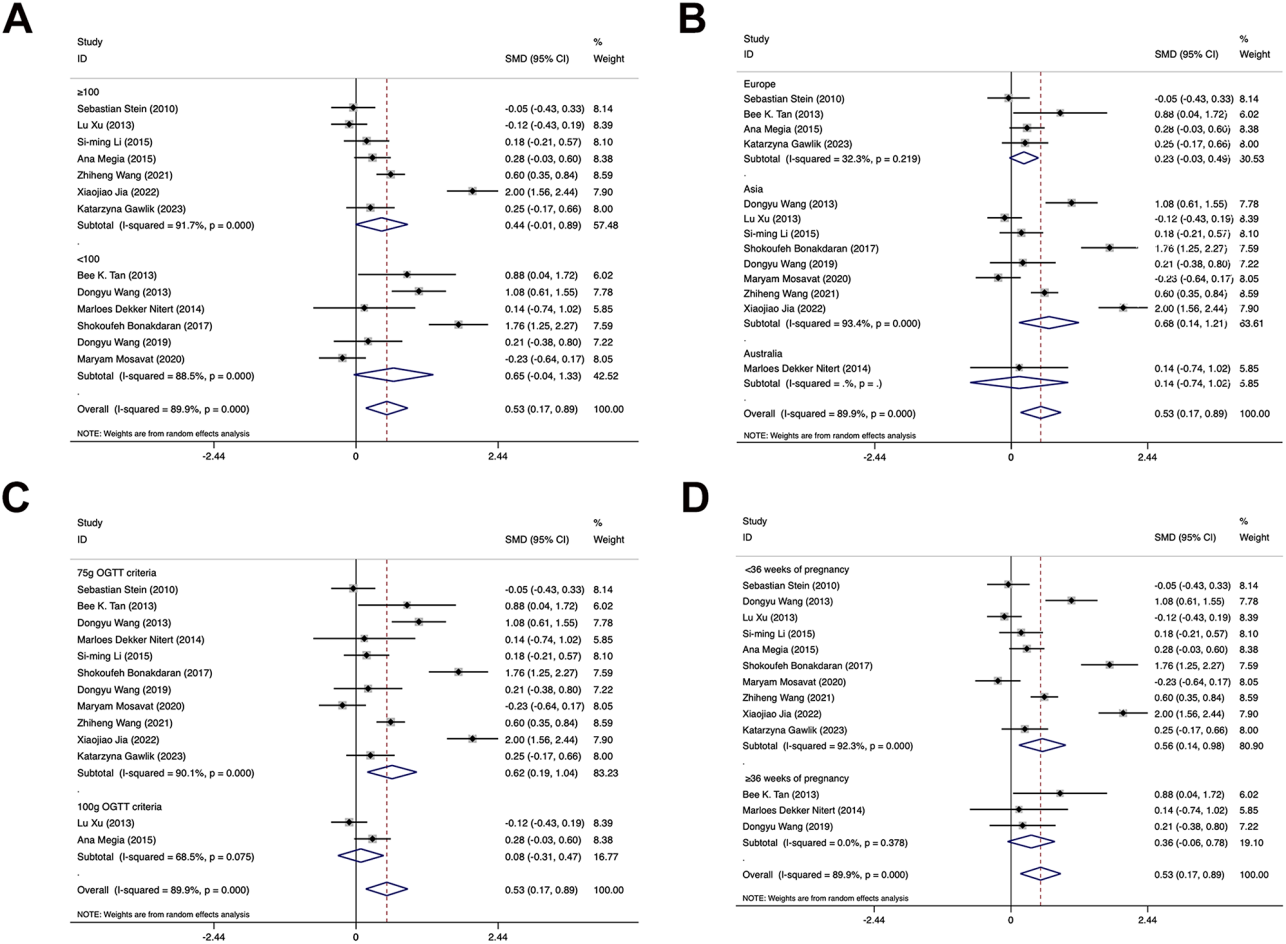
**A**: Forest plot of evaluating the relationship between FGF21 levels and GDM in the subgroup analysis by sample size. SMD: Standard Mean Difference; CIs: Confidence Intervals. **B**: Forest plot of investigating the relationship between FGF21 levels and GDM in the subgroup analysis by region. SMD: Standard Mean Difference; CIs: Confidence Intervals. **C**: Forest plot of investigating the relationship between FGF21 levels and GDM in the subgroup analysis by diagnostic criteria. SMD: Standard Mean Difference; CIs: Confidence Intervals. **D**: Forest plot of investigating the relationship between FGF21 levels and GDM in the subgroup analysis by the time point of sample collection. SMD: Standard Mean Difference; CIs: Confidence Intervals

Subgroup analyses were executed based on the sample size of the included studies, region, diagnostic criteria for GDM and the time of sample collection. The results revealed that the region of Europe (*I*^2^ = 32.3%, *p* = 0.022) and the time of sample collection of ≥ 36 weeks (*I*^2^ = 0%, *p* = 0.000) significantly decreased heterogeneity, and there were no marked effects of other factors including the sample size, diagnostic criteria for OGTT on heterogeneity, as detailed in Table 2; Fig. 4. Subgroup analyses by sample size yielded similar results, indicating that circulating FGF21 levels did not significantly differ between GDM-affected pregnant women and healthy expectant mothers (*p1* = 0.065, *p2* = 0.055). Results of subgroup analyses by region showed that, in the Asian group, GDM-affected pregnant women have significantly increased circulating FGF21 levels compared to healthy expectant mothers (*p* = 0.013), while in the European group, no obvious differences were observed in FGF21 levels between the case group and the control group (*p* = 0.085). Results of subgroup analyses by diagnostic criteria demonstrated that, in the group based on 75 g OGTT criteria, GDM-affected pregnant women had considerably greater levels of circulating FGF21 than healthy expectant mothers (*p* = 0.004), but in the group based on 100 g OGTT criteria, FGF21 levels did not significantly differ between the case and control groups (*p* = 0.684). Results of subgroup analyses by the time of sample collection manifested that, if sampled before 36 weeks of pregnancy, GDM-affected pregnant women had significantly higher levels of circulating FGF21 than healthy expectant mothers (*p* = 0.009), whereas if sampled at or after 36 weeks of pregnancy, FGF21 levels of the case group and the control group did not significantly differ from each other (*p* = 0.094).

**Table 2:** Subgroup analysis of FGF21 levels in healthy control pregnant women and pregnant women with GDM

Subgroup	Number of studies	SMD(95%CI)	*p*	Test(s) of heterogeneity: I^2^
**Sample size**				
< 100 ≥ 100	6 7	0.645 (-0.040, 1.330) 0.441 (-0.009, 0.891)	0.065 0.055	88.5% 91.7%
**Region**				
Asia Europe Australia	8 4 1	0.677 (0.140, 1.214) 0.228( -0.032, 0.487) -	0.013 0.085 -	93.4% 32.3% -
**Diagnostic criteria**				
75 g OGTT criteria 100 g OGTT criteria	11 2	0.620 (0.194, 1.045) 0.082 (-0.312, 0.475)	0.004 0.684	90.1% 68.5%
**Time point of sample collection**				
< 36 weeks of pregnancy ≥ 36 weeks of pregnancy	10 3	0.561( 0.142, 0.981) 0.362( -0.061, 0.785)	0.009 0.094	92.3% 0.0%
**Overall**	13	0.529 (0.168, 0.890)	0.004	89.9%

Meta-regression analysis was employed to examine the influence of the age and body mass index within the case group on effect sizes. The results showed that the age of the case group was a significant factor affecting the heterogeneity (meta regression coefficient: -0.209; 95%CI: -0.405 ~ -0.014; *p* = 0.038), while body mass index had no significant effect on heterogeneity (meta-regression coefficient: -0.012; 95%CI: -0.124 ~ 0.100; *p* = 0.809).

**Fig. 5:**
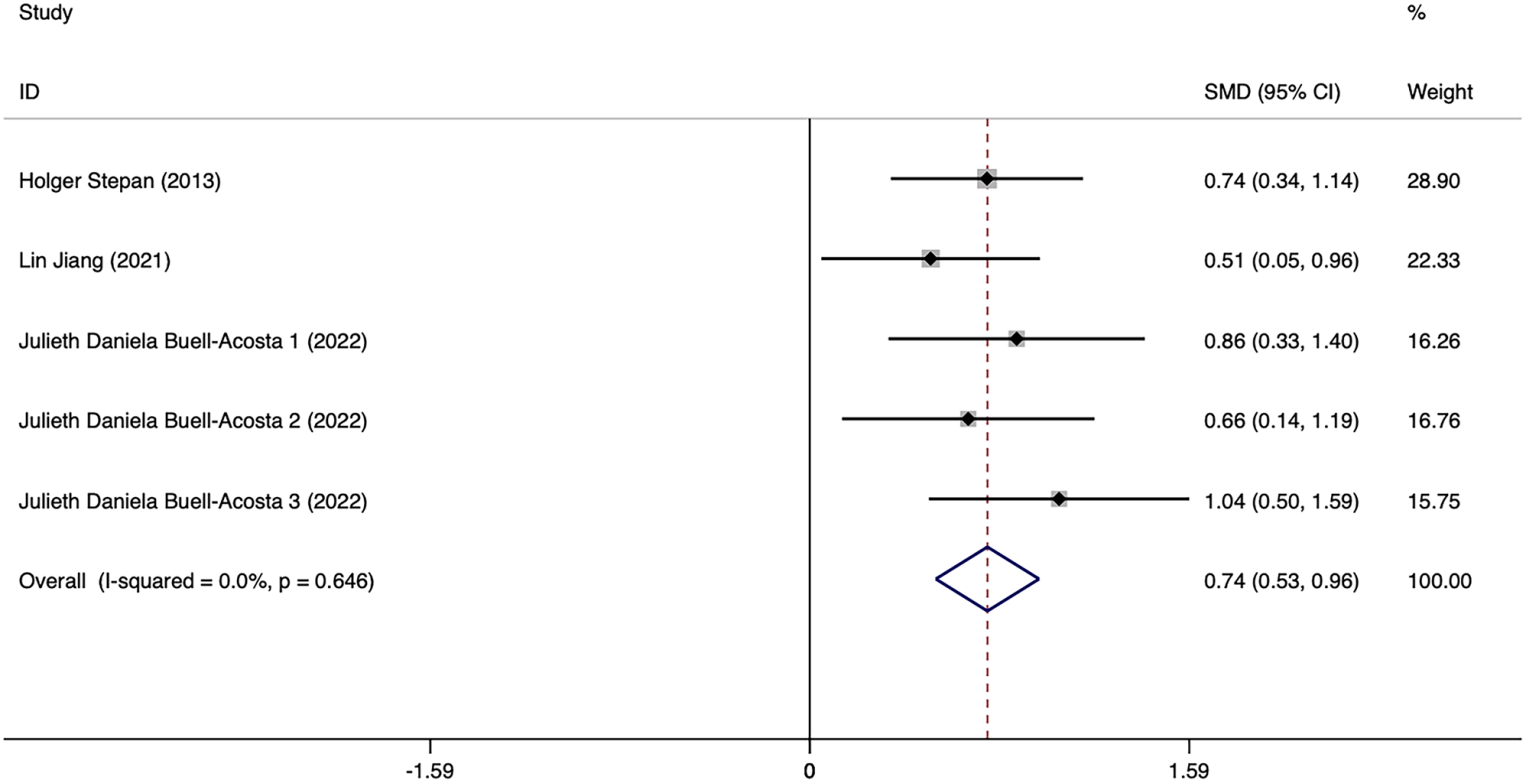
Forest plot of meta-analysis of the relationship between FGF21 levels and PE. SMD: Standard Mean Difference; CIs: Confidence Intervals

**Fig. 6:**
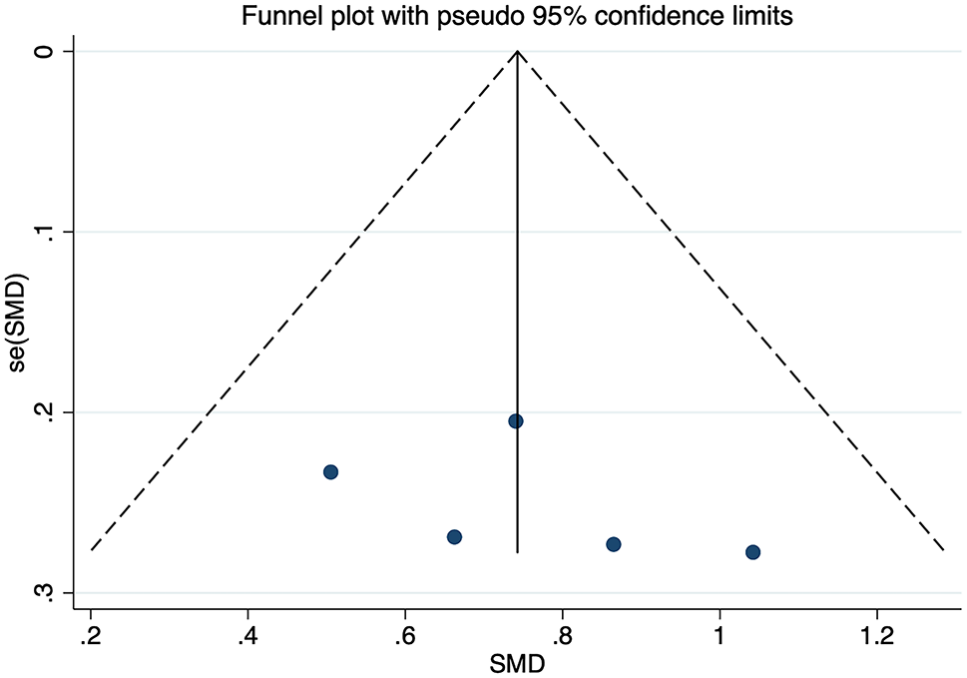
Funnel plot of publication bias for FGF21 levels and PE. SMD: Standard Mean Difference; se(SMD): Standard Error of Standard Mean Difference

Three studies related to PE included 120 pregnant women with PE and 134 healthy expectant mothers. Among them, one study covered three separate sets of data obtained in the first, second and third trimesters. Results showed no significant heterogeneity among five sets of data (*I*^2^ = 0%, *p* = 0.000) (Fig. 5). For data analysis, a fixed-effects model was employed. The results revealed that pregnant women with PE exhibited significantly elevated levels of circulating FGF21 than healthy expectant mothers (SMD = 0.743, 95% CI: 0.527 ~ 0.958). The differences were statistically significant (*p* = 0.000). Sensitivity analyses were carried out by excluding one study at a time, and the results demonstrated that none of the individual studies significantly changed the effect size. Begg’s and Egger’s tests were used to determine if publication bias existed, and results indicated that the funnel plot was approximately symmetrical, suggesting that studies included in the meta-analyses did not exhibit any notable publication bias (*t* = 0.92, *p* = 0.426), as shown in Fig. 6. No further subgroup analyses were performed as the number of studies was relatively small.

## Discussion

GDM, one of the most frequent complications during pregnancy, is connected with multiple adverse pregnancy outcomes, and has grown to be a worldwide public health concern with the prevalence of overweight, obesity and delayed childbearing. An OGTT test performed in the second and third trimester of pregnancy is the widely-used screening method for GDM. However, there is no globally unified standard yet, and a series of complicated standardized processes requires the cooperation of pregnant women. PE and GDM share similar risk factors as well as pathophysiological changes. PE, a major complication of pregnancy, is one of the primary causes of maternal mortality. However, traditional diagnostic methods are deficient in terms of their sensitivity and specificity. Therefore, discovering biomarkers related to GDM or PE is of great clinical significance and has received extensive attention from scholars around the world [15–17].

Energy homeostasis and glycolipid metabolism are significantly maintained and regulated by FGF21, a multifunctional protein that is primarily released by adipose tissue, liver and pancreas, plays a significant part in regulating glycolipid metabolism and maintaining energy homeostasis, and is closely associated with many chronic diseases [11, 18–20]. It has been shown that FGF21 improves insulin sensitivity, insulin resistance and β-cell function, and exerts a protective effect through alleviating ROS-mediated oxidative stress, inflammation and apoptosis [21–24]. A large-scale prospective study conducted by Chen et al. found that FGF21 levels steadily rose as the blood sugar disorder deteriorated, and concluded that FGF21 can predict the occurrence of type 2 diabetes [25]. Mosavat et al. performed a longitudinal case-control study and reported patients with GDM exhibited notably higher FGF21 levels compared to healthy controls [26]. Current research suggests that elevated serum FGF21 levels in patients with type 2 diabetes and GDM may result from the body’s compensatory mechanism. As the expression of FGF receptor 1 and β-klotho receptor in white adipocytes decreased, FGF21 cannot bind properly to form co-receptors, resulting in the compensatory increase in circulating FGF21 levels. On the other hand, due to metabolic disorders and reduced sensitivity to FGF21, the liver must compensatorily synthesize and secrete more FGF21 to maintain metabolic homeostasis [27, 28]. FGF21 can promote insulin secretion by enhancing the expression of insulin gene transcription factors and N-ethylmaleimide-sensitive factor attachment protein receptor protein, thereby activating the phosphatidylinositol 3-kinase/Akt signaling pathway [29]. Moreover, Stepan et al. discovered that serum FGF21 levels in patients with PE were nearly three times greater than those in healthy expectant mothers, and they decreased to levels close to those of healthy controls after 6 months of pregnancy [30]. However, the physiological mechanism associated with elevated FGF21 levels in pregnant women with PE remains unclear. Some studies have suggested that FGF21 can suppress hypertension and reduce vascular damage by converting angiotensin II to angiotensin-(1–7) [31]. Is this physiological mechanism also applicable to PE? Is the increased FGF21 in PE patients also a compensatory mechanism? These questions require further exploration through additional studies. As the understanding of the correlation between FGF21 levels and GDM and PE deepened in recent years, we conducted meta-analyses on published articles based on the above foundation, to figure out the value of FGF21 levels in diagnosing GDM and PE.

The analysis results displayed that those GDM- and PE-affected pregnant women presented noticeably higher FGF21 levels compared to healthy expectant mothers. Moreover, FGF21 levels in pregnant women and the occurrence of GDM and PE are strongly related. Due to significant heterogeneity between was observed in the research on the correlation between FGF21 and the risk of GDM, subgroup analyses were thus performed and indicated that the factors affecting heterogeneity were the region where a study performed and the time of sample collection. Results of subgroup analyses manifested that, if sampled before 36 weeks of pregnancy, compared to healthy expectant mothers, FGF21 levels were significantly greater in pregnant women (*p* = 0.009), whereas if sampled at or after 36 weeks of pregnancy, outcomes of the two groups showed no statistically significant difference (*p* = 0.094). Referring to the study by Sutton, E. F. et al., in healthy pregnant women, FGF21 levels increased with advancing gestational age. Compared to the first trimester, the FGF21 levels in the third trimester were generally more than twice as high [32]. We speculate that the possible reason is that FGF21 levels increased to a greater extent in pregnant women with GDM than in healthy pregnant women before 36 weeks of pregnancy, resulting in a significant difference between the two groups. After 36 weeks of pregnancy, the FGF21 levels in healthy pregnant women increased at a greater rate than those in pregnant women with GDM, thereby reducing the difference between the two groups. More research is needed in the future to validate the above hypotheses and to explore changes in FGF21 levels throughout pregnancy in the GDM case group and control group. This will facilitate a deeper understanding of the role of FGF21 in the development and progression of the disease. Data from the European and Asian subgroups yielded different results. Specifically, in the Asian group, GDM-affected pregnant women presented with markedly higher circulating FGF21 levels compared to healthy expectant mothers (*p* = 0.013), however, in the European group, there was no obvious distinction between the two groups (*p* = 0.085). More studies from different regions need to be included in the future to further explore whether the significantly increased FGF21 levels only can predict GDM in Asian women.

The results of a previously published meta-analysis study by Jue Jia et al. on FGF21 levels in GDM-affected pregnant women pointed out that [33], compared to healthy controls, FGF21 levels were significantly higher in GDM-affected pregnant women (95%CI: 0.07 ~ 0.86, *p* = 0.02), which yielded similar results with this study. In this study, we included an additional 1 GDM-related study published in 2020, 1 in 2021, 1 in 2022, and 1 in 2023 [26, 34–36] based on the research by Jue Jia et al., and we also added 3 studies related to PE [30, 37, 38] to further supplement recent data and expand the sample size. To our knowledge, this meta-analysis examined the relationship between FGF21 levels and two disorders (GDM and PE) for the first time.

Although there are some strengths compared to individual studies, limitations still exist. First, the scope of the research data retrieval is limited, encompassing restricted coverage of databases and languages. Second, in addition to the factors examined in the subgroup analysis and meta-regression analysis (sample size, region, diagnostic criteria for GDM, timing of sample collection, age, body mass index), potential confounders such as the health-related behaviors (diet, exercise, etc.), physical examinations and biochemical indicators (blood lipid levels, etc.) may affect the levels of the metabolic indicator FGF21. Since there were inadequate detailed data in the original studies, we did not explore the impact of other factors. Third, the above conclusions need further verification and improvement in the future, as the number of existing studies and the involved sample size are relatively small. On one hand, there is limited research data on early pregnancy, and more research efforts are needed. If there is potential for enabling early diagnosis, it will be of great significance for clinical diagnosis and early intervention. On the other hand, more studies are needed to continuously monitor changes in FGF21 levels from early pregnancy, which will help us further understand the correlation between FGF21 and the development and progression of diseases.

Studies on diagnostic markers for GDM or PE have been conducted more often in recent years. Previous studies indicated that some adipocyte derivatives and inflammatory markers, including insulin-like growth factor-1, adiponectin and leptin, also have the potential to be biomarkers for its diagnosis to some extent [39–41], but concerns still existed, for instance, different conclusions, poor specificity or sensitivity. We believe that the construction of novel diagnostic models with biomarker panels that demonstrate favorable diagnostic potential might obtain better diagnostic efficacy, and more studies are required to explore this further. In addition, studies have found that when FGF21 was used to treat mice fed a high-fat diet, insulin resistance was significantly improved. Overexpression of FGF21 in the liver upregulated the expression of genes involved in fatty acid oxidation, accelerated energy consumption, and reduced fatty degeneration, all of which were beneficial for the treatment of diabetes [42, 43]. Whether FGF21 has the potential to be used in the treatment of hyperglycemia also requires further exploration.

## Conclusion

Our research showed that FGF21 levels were certainly higher in pregnant women with PE and GDM than in healthy expectant mothers throughout the same period. Due to the limited number of studies and the fact that most data were derived from the second and third trimesters of pregnancy, additional research is necessary to validate the conclusions, investigate the potential of FGF21 in enabling early diagnosis, and further examine the role of FGF21 in the development and progression of GDM/PE.

## Electronic supplementary material

Below is the link to the electronic supplementary material.

Supplementary Material 1: Search strategy

Supplementary Material 2: Quality assessment based on the Newcastle-Ottawa Scale of studies included in this meta-analysis

## Data Availability

The datasets generated during and/or analysed during the current study are available from the corresponding author on reasonable request.

## Abbreviations

FGF21: Fibroblast growth factor 21
GDM: Gestational diabetes mellitus
PE: Preeclampsia
SMDs: Standardized mean differences
CIs: Confidence intervals
OGTT: Oral glucose tolerance test
SD: Standard deviation
SMDs: Standardized mean differences
