# Supplementary material for: Circulating fibroblast growth factor 21 levels in gestational diabetes mellitus and preeclampsia: a systematic review and meta-analysis
Source: BMC Pregnancy Childbirth. 2025 Jan 16;25:34. doi: 10.1186/s12884-025-07157-3 (PMC11740615; doi:10.1186/s12884-025-07157-3)
Supplement: Supplementary file 1 — Supplementary Material 1: Search strategy [file 12884_2025_7157_MOESM1_ESM.docx]

**Supplementary material 1 Search strategy**

PubMed search formula

# 1) “Search ("Diabetes, Gestational"[Mesh]) OR (((((((Diabetes,

Gestational[Title/Abstract]) OR (Diabetes, Pregnancy-Induced[Title/Abstract])) OR (Diabetes, Pregnancy Induced[Title/Abstract])) OR (Pregnancy-Induced

Diabetes[Title/Abstract])) OR (Gestational Diabetes[Title/Abstract])) OR (Diabetes

Mellitus, Gestational[Title/Abstract])) OR (Gestational Diabetes Mellitus[Title/Abstract]))

#2) “Search ("Pre-Eclampsia"[Mesh]) OR (((((((((((((((((((((((((((((((((((Pre- Eclampsia[Title/Abstract]) OR (Pre Eclampsia[Title/Abstract])) OR

(Preeclampsia[Title/Abstract])) OR (Pregnancy Toxemias[Title/Abstract])) OR

(Pregnancy Toxemia[Title/Abstract])) OR (Toxemia, Pregnancy[Title/Abstract])) OR (Edema-Proteinuria-Hypertension Gestosis[Title/Abstract])) OR (Edema Proteinuria Hypertension Gestosis[Title/Abstract])) OR (Gestosis, Edema-Proteinuria-

Hypertension[Title/Abstract])) OR (Hypertension-Edema-Proteinuria Gestosis[Title/Abstract])) OR (Gestosis, Hypertension-Edema-

Proteinuria[Title/Abstract])) OR (Hypertension Edema Proteinuria

Gestosis[Title/Abstract])) OR (Toxemia Of Pregnancy[Title/Abstract])) OR (Of

Pregnancies, Toxemia[Title/Abstract])) OR (Of Pregnancy, Toxemia[Title/Abstract])) OR (Pregnancies, Toxemia Of[Title/Abstract])) OR (Pregnancy, Toxemia

Of[Title/Abstract])) OR (Toxemia Of Pregnancies[Title/Abstract])) OR (EPH Complex[Title/Abstract])) OR (EPH Toxemias[Title/Abstract])) OR (EPH

Toxemia[Title/Abstract])) OR (Toxemia, EPH[Title/Abstract])) OR (Toxemias, EPH[Title/Abstract])) OR (EPH Gestosis[Title/Abstract])) OR (Gestosis,

EPH[Title/Abstract])) OR (Toxemias, Pregnancy[Title/Abstract])) OR (Preeclampsia Eclampsia 1[Title/Abstract])) OR ( 1, Preeclampsia Eclampsia[Title/Abstract])) OR ( 1s, Preeclampsia Eclampsia[Title/Abstract])) OR (Eclampsia 1,

Preeclampsia[Title/Abstract])) OR (Eclampsia 1s, Preeclampsia[Title/Abstract])) OR (Preeclampsia Eclampsia 1s[Title/Abstract])) OR (Proteinuria-Edema-Hypertension

Gestosis[Title/Abstract])) OR (Gestosis, Proteinuria-Edema-

Hypertension[Title/Abstract])) OR (Proteinuria Edema Hypertension Gestosis[Title/Abstract]))

#3) “Search (((((fibroblast growth factor 21[Title/Abstract]) OR (FGF-21[Title/Abstract])) OR (FGF21[Title/Abstract])) OR (fibroblast growth factor 21 protein[Title/Abstract])) OR (FGF21 protein[Title/Abstract])) OR (FGF-21 protein[Title/Abstract])

#4) “Search #1 OR #2 #5) “Search #4 AND #3
