# Supplementary material for: Circulating fibroblast growth factor 21 levels in gestational diabetes mellitus and preeclampsia: a systematic review and meta-analysis
Source: BMC Pregnancy Childbirth. 2025 Jan 16;25:34. doi: 10.1186/s12884-025-07157-3 (PMC11740615; doi:10.1186/s12884-025-07157-3)
Supplement: Supplementary file 2 — Supplementary Material 2: Quality assessment based on the Newcastle-Ottawa Scale of studies included in this meta-analysis [file 12884_2025_7157_MOESM2_ESM.docx]

**Supplementary material 2.** Quality assessment based on the Newcastle-Ottawa Scale of studies included in this meta-analysis

| **Author, year** | **Selection** | | | | **Comparability** | **Exposure** | | | **Score** |
| --- | --- | --- | --- | --- | --- | --- | --- | --- | --- |
|  | **An adequate**  **definition of**  **case** | **Representative**  **ness of the case** | **Selection of**  **controls** | **Definition of**  **controls** | **Control for an**  **important factor** | **Assessment of**  **exposure** | **The same method of**  **ascertainment for cases**  **and controls** | **Non-response**  **rate** |  |
| Sebastian Stein *et al.*, 2010 | ★ | ★ | – | ★ | – | ★ | ★ | ★ | 6 |
| Holger Stepan *et al.*, 2013 | ★ | ★ | – | ★ | ★★ | ★ | ★ | ★ | 8 |
| Bee K. Tan *et al.*, 2013 | ★ | ★ | – | ★ | ★★ | ★ | ★ | ★ | 8 |
| Dongyu Wang *et al*., 2013 | ★ | ★ | – | ★ | ★★ | ★ | ★ | ★ | 8 |
| Lu Xu *et al*., 2013 | ★ | ★ | – | ★ | ★★ | ★ | ★ | ★ | 8 |
| Marloes Dekker Nitert *et al*., 2014 | ★ | ★ | – | ★ | – | ★ | ★ | ★ | 6 |
| Si-ming Li *et al.*, 2015 | ★ | ★ | – | ★ | ★★ | ★ | ★ | ★ | 8 |
| Ana Megia *et al*., 2015 | ★ | ★ | – | ★ | ★★ | ★ | ★ | ★ | 8 |
| Shokoufeh Bonakdaran *et al.*, 2017 | ★ | ★ | – | ★ | ★★ | ★ | ★ | ★ | 8 |
| Dongyu Wang *et al*., 2019 | ★ | ★ | – | ★ | ★ | ★ | ★ | ★ | 7 |
| MaryamMosavat *et al.*, 2020 | ★ | ★ | – | ★ | ★★ | ★ | ★ | ★ | 8 |
| Lin Jiang *et al.*, 2020 | ★ | ★ | – | ★ | ★★ | ★ | ★ | ★ | 8 |
| Zhiheng Wang *et al*., 2021 | ★ | ★ | – | ★ | ★★ | ★ | ★ | ★ | 8 |
| Julieth Daniela Buell-Acosta *et al.*, 2022 | ★ | ★ | – | ★ | ★ | ★ | ★ | ★ | 7 |
| Xiaojiao Jia *et al*., 2022 | ★ | ★ | – | ★ | ★ | ★ | ★ | ★ | 7 |
| Katarzyna Gawlik *et al*., 2023 | ★ | ★ | – | ★ | ★ | ★ | ★ | ★ | 7 |
